# Identification and Characterization of Fiber Optic Imaging Bundle Structures in Endoscopic Fringe Projection Systems

**DOI:** 10.3390/s25113305

**Published:** 2025-05-24

**Authors:** Jannis Drangmeister, Markus Kästner, Eduard Reithmeier

**Affiliations:** Institute of Measurement and Automatic Control, Stiftung Gottfried Wilhelm Leibniz Universität Hannover, An der Universität 1, D-30823 Garbsen, Germany; markus.kaestner@imr.uni-hannover.de (M.K.); sekretariat@imr.uni-hannover.de (E.R.)

**Keywords:** endoscopic fringe projection, fiber optic imaging bundles, fringe pattern optimization

## Abstract

Endoscopic fringe projection is used to perform inspections of hard-to-reach areas. In order to transfer fringe patterns from a projector to the specimens’ surface, fiber optic imaging bundles (FOIB) can be employed. To ensure maximum accessibility, a highly flexible FOIB is needed. Therefore, the number of individual fibers has to be minimized, which affects the quality of the fringe pattern. This paper presents methods and results for projecting a high frequency pattern despite a small number of fibers by adapting the FOIBs’ structure. First, the spatial structure of the FOIB is identified with regard to the projector pixels. By determining their center, it is possible to address individual fibers. It will be shown that the peak values of spots produced by individual fibers behave nonlinearly according to the modulated intensity. Furthermore, the intensity distribution within the spots changes. By recording the intensity curves, the presented algorithm is able to adapt the fringe pattern in orientation and intensity. This leads, especially for high frequency patterns, to an improved amplitude and signal-to-noise ratio.

## 1. Introduction

Endoscopic fringe projection systems have been utilized for the three-dimensional reconstruction of otherwise inaccessible areas, as well as to facilitate the detection of defects. The underlying principle is analogous to that of classic fringe projection [1]. By projecting fringe patterns on the specimen surface, and detecting them from a different viewing angle, surface points are reconstructed by triangulating outgoing camera and projector rays. To assign the correct projector pixel on the specimen surface, the camera has to unwrap the phase information in the fringe pattern. Since space is a restricted factor in endoscopic systems, special extensions are utilized in this instance. Endoscopic systems can be categorized into two distinct types: rigid and flexible measuring systems. In rigid systems, lens-based endoscopes are employed to transmit the image or the fringe pattern [1]. In flexible systems, both the pattern and the camera image can be transmitted by an FOIB [2]. Alternatively, a chip-on-tip camera is used instead of an additional FOIB [3]. This is also the design on which this work is based.

Flexible endoscopes have the advantage of better accessibility. A disadvantage of using an FOIB pertains to its impact on the transmitted pattern. In the literature, FOIBs produced by Fujikura are widely used [2,3,4,5,6]. These are silica-based coherent fiber bundles with a number of single fibers in a range from 1600 to 100,000, which are arranged in a hexagonal shape [7]. As the fibers are oriented in parallel, the exchange of light between them is possible. This phenomenon is also referred as crosstalk, and its strength is heavily dependent on the internal structure of the FOIB. As shown in [4], the polished extremity of an FOIB captured by a scanning electron microscope features not a perfectly hexagonal, but rather a semi regular structure, which results from the production process. Additionally, single fibers vary in size and orientation, and as a consequence, also in brightness. However, this irregular structure has the advantage of reducing crosstalk. This assertion is substantiated through theoretical analyses. The precise transmission behavior is determined by a complex interaction between the opto-geometric characteristics of the FOIB [8].

A different type of FOIB are leached fibers, inter alia manufactured by Schott. The individual fibers in this type are not fused along their entire length, but only at the ends, which leads to much higher flexibility [5]. In addition, the structure is much more homogeneous compared to the image guides from other manufacturers. Despite the regular structure, these imaging bundles do not suffer from strong crosstalk [9].

In relation to fringe projection, the FOIB can affect both low-frequency [10] and high-frequency patterns [6]. Especially in the case of high frequency patterns, the limited number of fibers can lead to problems, as only one intensity is transmitted per fiber. If the frequency is high enough, the pattern overlaps with the structure of the FOIB, and fringes become unrecognizable on the specimen surface [11]. The latter effect is particularly apparent in highly flexible applications, as the image guides become more flexible as the number of fibers decreases [7].

Optimizing the pattern frequency in order to reduce measurement deviation has already been extensively investigated in classical fringe projection. For the measurement itself, high-frequency fringe patterns are used. The 2 pi periodic phase can be calculated using several shifts. Special algorithms or additional patterns are required to determine the absolute phase [12]. This means that the actual pixel information is contained in the high-frequency patterns. There are several factors that can cause pixels to be misallocated. Every system is subject to noise, for example, from the camera or ambient light. Noise is particularly problematic when the signal amplitude decreases. This results from the lowpass behavior of optical systems [13]. Furthermore, projectors often have high apertures in order to achieve the highest possible brightness. This also leads to a low sharpness range and, as a result, to a lower amplitude outside the optimal focus [14]. The required optimal frequency is contingent upon system-specific parameters and the employed phase shift algorithm. However, certain fundamental principles can be outlined. In order to minimize the measurement uncertainty, it is imperative to set the frequency to a high value, given that this will enhance phase sensitivity and facilitate more precise projector pixel allocation. An analytical expression for the phase measurement uncertainty is formulated by the following equations for phase-shifting algorithms [15]:

(1) $$I_{n}\left(x,y\right)=A\left(x,y\right)+B\left(x,y\right)\cos\left[\Phi \left(x,y\right)-\frac{2\pi n}{N}\right]$$

(2) $$\sigma_{\Phi }^{2}\left(x,y\right)=\frac{2}{Nf^{2}}\cdot \frac{\sigma^{2}}{B^{2}}$$

Equation (1) provides a description of the ideal intensity distribution in the camera image. Each position (x,y) in the image is described by the background brightness *A* and the signal with the amplitude *B*, the wrapped phase Φ, and the shift index n=0,1,2,…,N−1. In consideration of the aforementioned relationship, Equation (2) quantifies the phase measurement uncertainty as a function of the number of shift steps *N*, the frequency of the pattern *f*, the noises standard deviation σ, and the signal amplitude *B* in the camera image. It is evident that the phase measurement uncertainty can be mitigated by the increasing frequency and signal amplitude, and by reducing the signal noise. Furthermore, an increasing number of shift steps exerts a positive effect.

The present work aims to reduce the measurement uncertainty by generating high-quality and high-frequency patterns, irrespective of the FOIB employed. To achieve this objective, the fringe pattern is adapted to the hexagonal structure of the FOIB. It will be demonstrated that, by aligning the fringe pattern to the structure and adjusting the brightness of individual fibers, both amplitude and signal-to-noise ratio are improved.

## 2. Materials and Methods

**Figure 1:**
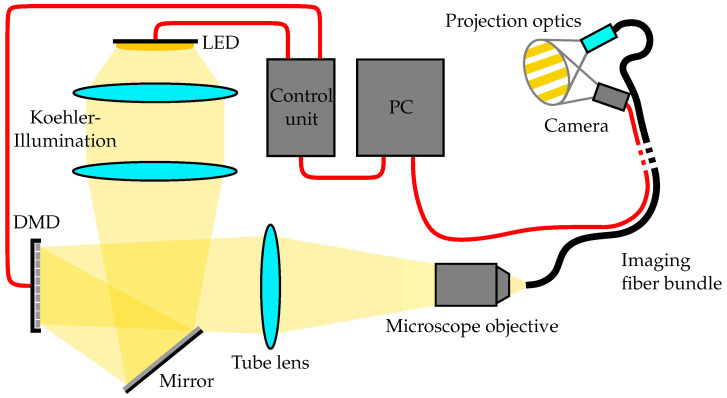
Schematic experimental setup.

**Figure A1:**
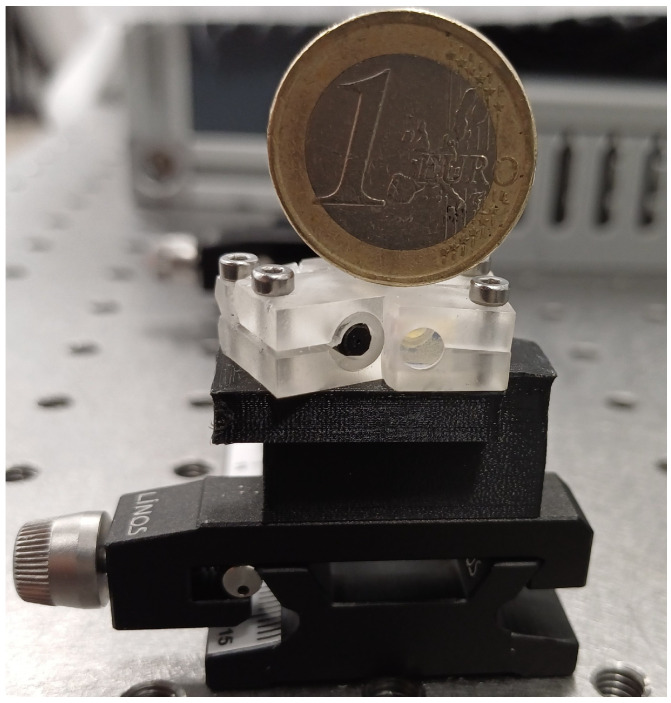
Arrangement of camera (left side) and projection optics (right side), which focuses on the image of the FOIB on a screen. The distance between the optical axes of the camera and projector is 6 mm, and the angle between them is 25°. To obtain a better idea of the overall size, a EUR 1 coin was placed on the sensor.

The experimental setup used in this work is illustrated in Figure 1. An image of the measuring head can be found in Appendix Figure A1. The projection unit contains a V-7001 digital micromirror device (DMD) (Vialux GmbH, Chemnitz, Germany) [16], which is illuminated by a 520 nm high power Ostar LED (Osram GmbH, Munich, Germany) [17] with connected Köhler illumination. Different projection intensities on the specimen are achieved by a pulse width modulation of the DMDs micromirrors in 8 bit resolution. The image generated by the DMD is coupled into the proximal end of the FOIB via a 125 mm tube lens and a 18 mm infinity corrected microscope objective (Olympus, Tokyo, Japan). The projection optics at the distal end consists of an infinity focusing GRIN lens and a plano convex lens with a focal length of 10 mm. Using these optics, a circle of 10 mm diameter on a projection screen located in a 10 mm distance is illuminated. The projected image is recorded by a 1/9^′′^ chip-on-tip camera with a Resolution of 1280 px × 720 px (MISUMI Electronics Corp., Taipei, China) [18] and transmitted to the computer via a USB interface. A simple paper-based target is used to analyze the projected images. The setup is protected from ambient light by an enclosure.

The FOIB that this work focuses on is a leached fiber consisting of 18,000 single fibers and an image circle diameter of 1.45 mm (Schott AG, Mainz, Germany). In a previous work [11], a FIGH-15-600N (Fujikura Ltd., Tokyo, Japan) fused silica FOIB with a fiber count of 15,000 was used. Their characteristics are summarized in Table 1.

As outlined in the introduction, the employment of FOIBs can yield adverse outcomes for the projected image, most notably at elevated fringe frequencies. This study aims to optimize the projected pattern via the adaptation of the projector image. The primary focus is delineated by two key points:

- The spatial structure of the fibers;
- The individual fiber brightness.

**Figure 2:**
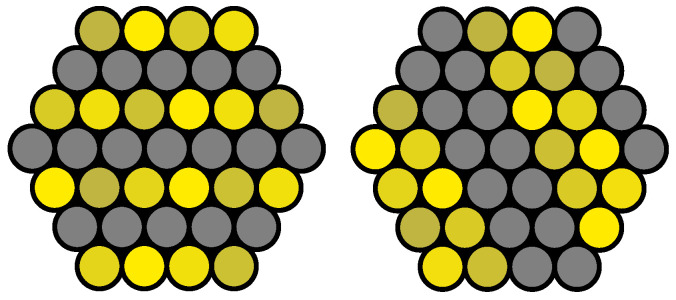
Examples for the adaption of fringe patterns. Grey means no illumination, while different shades of yellow represent different modulation intensities to equalize differences in fiber brightness.

As demonstrated in Figure 2, these two points can be utilized to enhance the quality of the pattern. First, the direction and phase of the fringe pattern are aligned with the layers in the hexagonal structure, provided that the structure is homogeneous. Figure 2 illustrates this adjustment for various frequencies, phases, and directions. At high frequencies, a binary pattern is produced, and at lower frequencies, the corresponding values of the cosine function have to be sampled.

Secondly, the intensity of the light projected into the fibers is adjusted. As mentioned in the introduction, individual fibers can vary in brightness. If this behavior is known, it is possible to adapt the modulated intensity in a way that the differences in brightness on the specimen surface are equalized, and consequently, the measurement uncertainty should be minimized.

The exact measurement uncertainty is contingent on the precise phase shift algorithm. Therefore, only the patterns themselves are evaluated. According to Equation (2), higher noise and a low signal amplitude in the pattern lead to higher phase measurement uncertainty. To assess the pattern quality in this scope, the signal-to-noise ratio (SNR) including the amplitude *B* and the standard deviation σ of the noise is used. As stated in [14], optical systems behave as a lowpass; therefore, not only high-frequency signals but also noise is suppressed. As a consequence, a high SNR does not necessarily lead to a high amplitude pattern which is robust to defocus and suitable for 3D reconstruction. For this reason, not only the SNR is evaluated, but also the amplitude itself. Setting the projected amplitude in relation to the modulated amplitude for different frequencies is also known as the modulation transfer function (MTF). Both SNR and MTF are commonly used in image processing [20].

To achieve the adaption, it is first of all essential to be aware of the precise correspondence between projector pixels and the individual fibers. It should be noted at this point, that, except for the measuring system itself, no additional material, e.g., cameras, was employed. This ensures a simple recalibration process of the system at any time. For demonstrative purposes, only a limited region of interest (ROI) of the projector containing 100 px × 100 px out of a total range of 768 px × 768 px was utilized initially.

**Figure 3:**
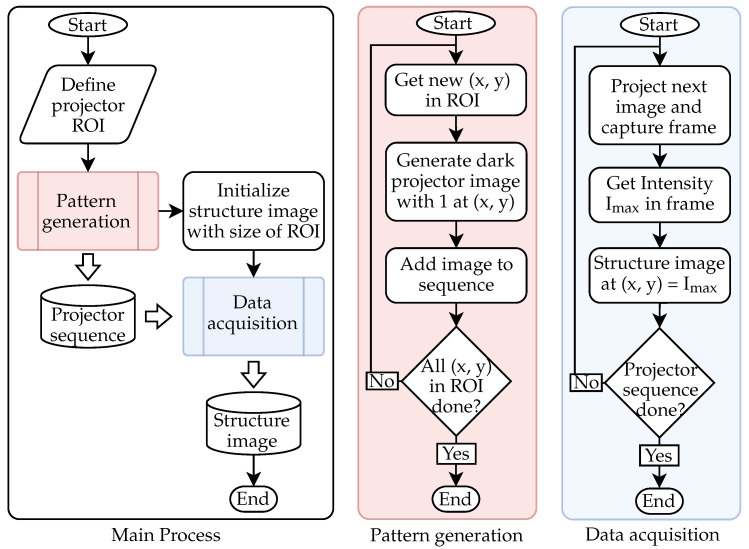
Process for identifying the FOIB structure.

Figure 3 shows the process of identifying the FOIB structure according to the projector. It was developed in [11], and provides robust identification even without focusing the FOIB on the projection screen. After defining the ROI in the center of the projector, the necessary projection sequence is generated. This consists of completely dark images, with only one bright pixel (corresponding to a value of 1.0) in each image at every position in the ROI. Before the data acquisition begins, an empty structure image with the size of the ROI is initialized. During the acquisition, every image of the projection sequence is displayed, while the camera detects the maximum intensity on the target. This intensity value is written according to the position of the bright projector pixel into the structure image. Projecting the bright pixel directly into a fiber leads to a high intensity peak on the screen. A pixel correlated to the cladding of a fiber produces a low intensity peak. In this manner, it is possible to observe the individual fibers of the FOIB.

**Figure 4:**
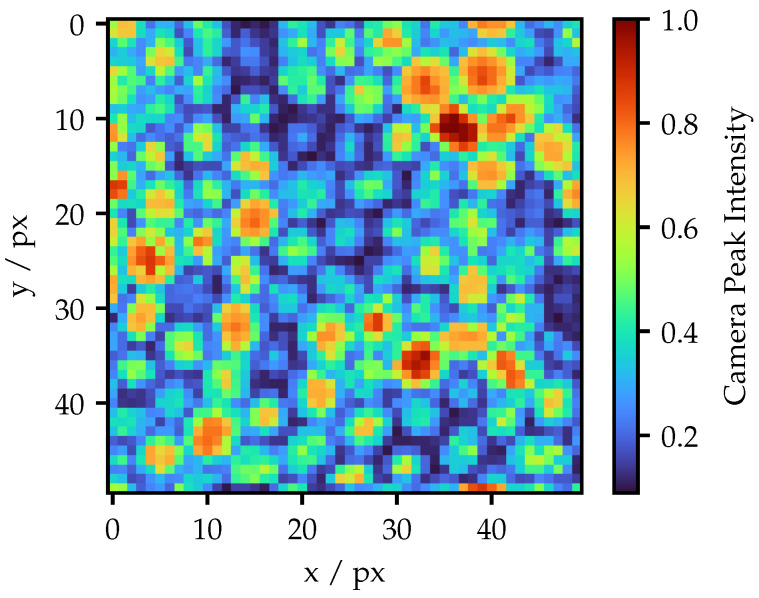
Outcome of the structure identification process for a Fujikura FOIB. All intensities are normalized to the maximum value.

An example result of the identification procedure of the Fujikura FOIB obtained in [11] is illustrated in Figure 4. Here, an ROI of 50 px × 50 px was utilized. Each peak represents a single fiber. It is clearly visible that the fibers vary in brightness and shape. The hexagonal structure is not recognizable in all areas, particularly in the upper middle section, where distinct fiber layers are interweaving. Since a homogeneous structure is required to adjust the fringe patterns according to Figure 2 on straight layers, the Fujikura FOIB is not suitable for this purpose. The results and further analysis, as well as the resulting adaption techniques of the Schott FOIB, are shown in the next section.

## 3. Results

### 3.1. Structure Identification

**Figure 5:**
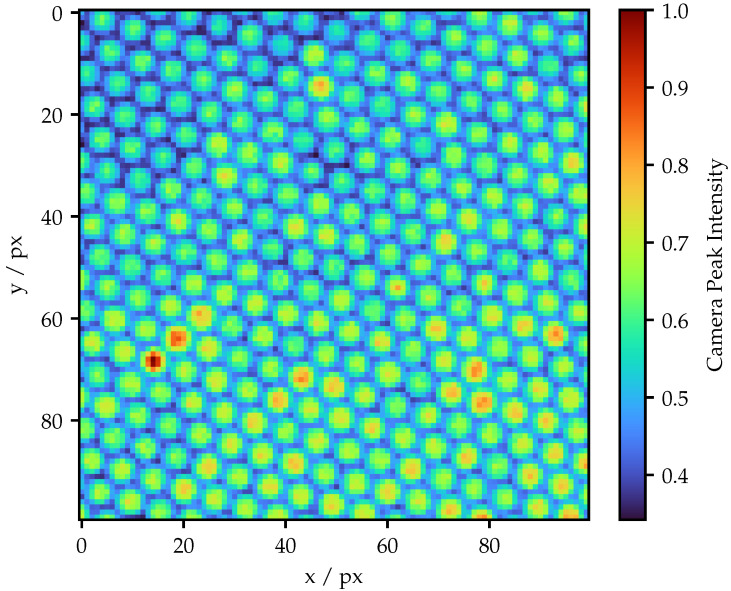
Structure image of the Schott FOIB. All intensities are normalized to the maximum value.

The result of the structure identification process of the Schott FOIB for a projector ROI of 100 px × 100 px is illustrated in Figure 5. The projection screen is located at a distance of approximately 10 mm, such that the projected ROI corresponds to an area of 1.3 mm × 1.3 mm. It is particularly apparent that the spatial structure is highly homogeneous compared to the Fujikura FOIB. Nevertheless, the individual fibers show significant differences in brightness.

**Figure 6:**
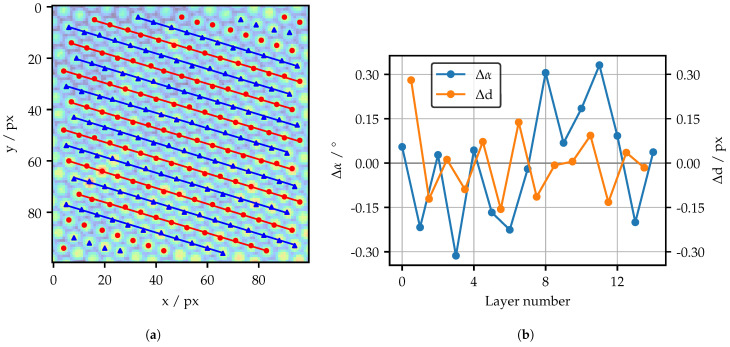
(**a**) Layer identification with determined peaks and fitted lines. (**b**) Analysis of the spatial structure.

**Figure A2:**
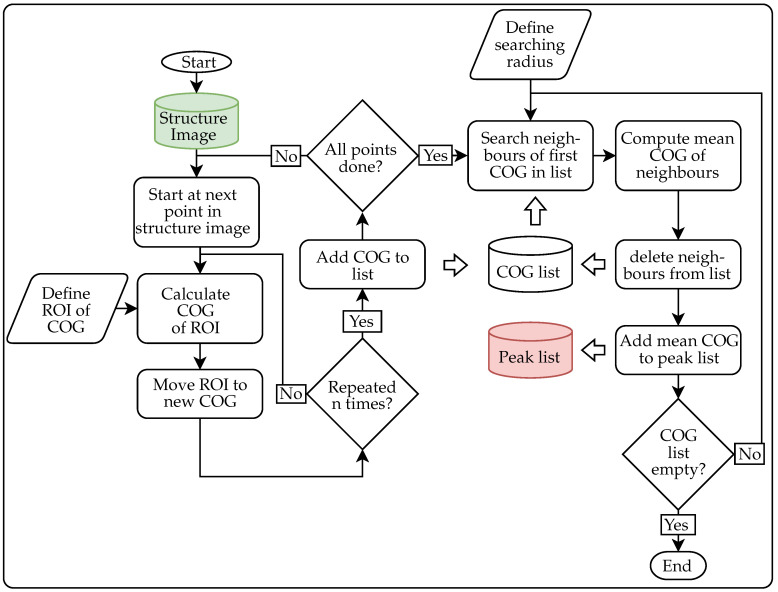
Algorithm for the peak determination. Starting at each image pixel, the adaptive center of gravity search converges to maxima in the image. In a second step, the identified positions are clustered. The mean position of the cluster is taken as center of the maxima.

A detailed analysis of the spatial structure is presented in Figure 6. Starting from the structure image, the positions of the local intensity maxima are determined first. This is achieved through the implementation of an adaptive center of gravity search starting at each pixel in the structure image. The precise algorithm is outlined in Appendix Figure A2. A simple binarization demonstrated limited robustness, primarily due to the substantial variation in the characteristics of the peaks, at least for the Fujikura FOIB.

Once the positions of the peaks are known, it is possible to determine the fiber layers. This is achieved by rotating the peak coordinates to an angle where the spread of their projected cluster on the y axis is minimized, meaning that the layers are aligned horizontally. By segmenting their cluster and rotating the coordinates back, the corresponding layer of each coordinate is known. As illustrated in Figure 2, there are three possible directions, each with an offset of 60 ° due to the hexagonal structure. It is therefore necessary to specify an angular range for the algorithm. In this work, only one direction is utilized.

Figure 6a illustrates the determined peaks, as well as the least-square-fitted lines through the peaks of the inner layers. As the fringe patterns are adapted to the structure, it is essential that the layers have a high degree of homogeneity, as measured by their spread in distance Δd and angle Δα. Therefore, the differences Δd and Δα from the orthogonal mean layer distance of 5.5 px and the mean layer angle of 16.8 ° are analyzed in Figure 6b. To avoid the influence of single outliers, the outer layers are not included.

The layer numbers are counted from the topmost layer downward. The maximum distance difference Δd does not exceed 0.3 px, while the angle difference Δα is marginally higher than 0.3°. In general, no consistent trend is observable. Thus, the structure only changes randomly on a small scale. Additionally, it is not always possible to determine the exact center of a fiber, due to the discrete nature of the projector pixel. Following these cognitions, it is concluded that the spatial structure of the Schott FOIB is sufficiently homogeneous to allow the adaptation of fringe patterns.

**Figure 7:**
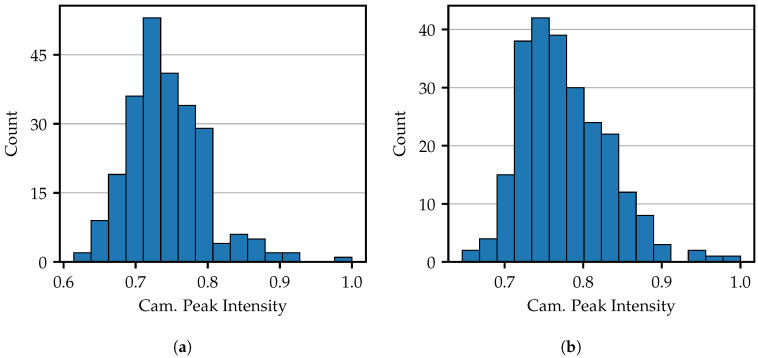
(**a**) Intensity distribution of the individual fibers with one projecting pixel per fiber and (**b**) 5 px projecting arranged in a cross.

As shown in Figure 5, the fibers exhibit varying levels of intensity. Figure 7 illustrates the distribution of the maximum brightness detected by the camera using the computed fiber centers. In Figure 7a, a single bright pixel is used per fiber, while Figure 7b utilizes five pixels arranged in a cross, offering a five-fold illumination area in a compact shape. These projection areas will also be used in further work. In both Figures, all values have been normalized to their maximum obtained value. One can see that the peak values are scattered in a range between 0.6 and 1.0. There is a significantly wider spread and more outliers particularly towards high intensity values. Comparing the 1 px and 5 px projection areas, it is noticeable that the overall distribution is shifted towards high intensities.

### 3.2. Peak Characterization

Up to this point, only the peak intensity values of the spots generated by individual fibers recorded by the camera have been examined. The subsequent section will address the precise characteristics of the spots in relation to the camera. To ensure the comparability between the different fibers as well as between the projection areas, all measurement series are recorded with the same camera exposure time and LED power. All camera intensities in this subsection are normalized to the maximum achieved intensity by the maximal bright fiber at a modulation of 1.0.

**Figure 8:**
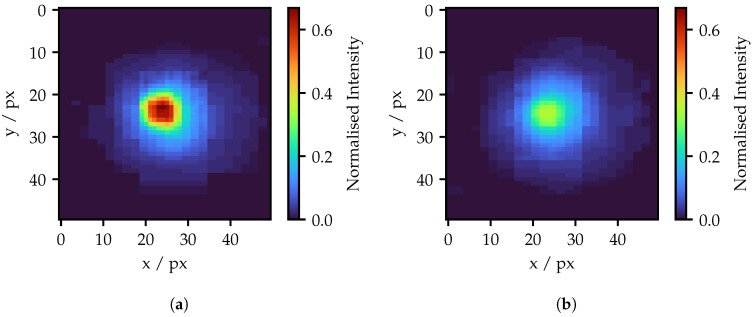
(**a**) Spot in the camera image produced by the maximal bright fiber and (**b**) the spot of the minimal bright fiber, both with a 5 px projection area and a modulated intensity of 0.45. The diameter of the spots does not exceed 40 px. This refers to a size on the projection screen of approximately 0.45 mm.

Figure 8 illustrates two examples of spots generated by individual fibers using a 5 px projection area. Both images are taken with a projector modulation of 0.45. On the left is the spot of the maximum bright fiber, on the right of the minimal bright fiber. Both examples will be taken up again later. Initially, both spots appear to have a Gaussian-like profile, while the peak values are exceeding a perspicuous difference in brightness. No signs of crosstalk are visible, like surrounding exited fibers.

**Figure 9:**
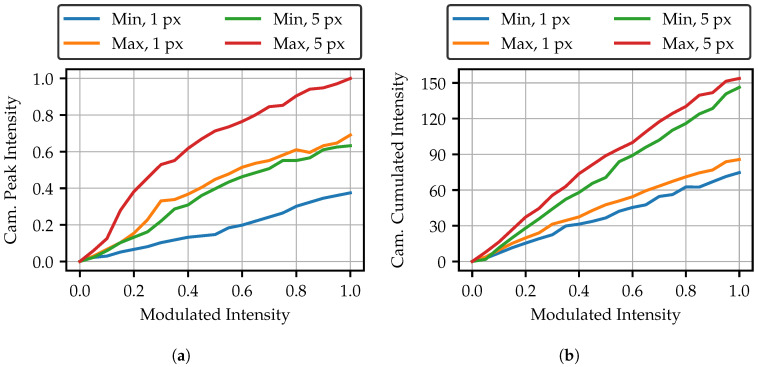
(**a**) Peak intensity of the fiber spots for increasing the modulated intensity. “Min” means minimal bright fiber, “Max” means maximal bright fiber. and (**b**) Overall sum of all pixels in the camera image for each spot.

The adapted fringe patterns will later consist of the superposition of several spots. To ensure a low noise pattern, the peak values of the spots in the same layer should be identical. As shown in Figure 2, this is achieved by modulating the fibers with different intensities. It is therefore of interest to know how the peak intensity behaves as a function of the projector modulation. It might be expected that the peak intensity of the spot would increase linearly, as the modulation only changes the time span during which the spot is displayed. But, as Figure 9a shows, the behavior of the peak intensity of the spots is highly nonlinear. Basically, a 5 px projection area allows a higher brightness. It seems not to make a difference to the shape of the curve whether 1 px or 5 px projection is used. This can be clearly seen through the curves of the maximum bright fiber with 1 px and the minimum bright fiber with a 5 px projection area. The shape of the curve only seems to depend on the maximum brightness of the fiber, since the lowest curve also suffers the least from curvature.

**Figure 10:**
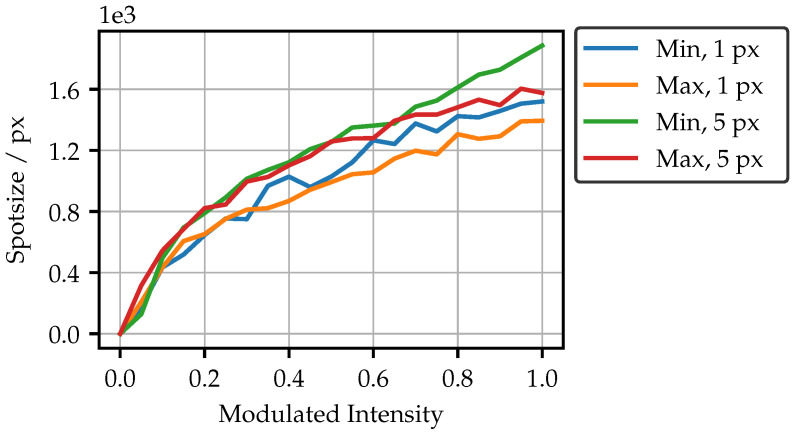
Spotsize to modulated intensity, as determined by a number of pixels with a value greater than zero.

As the peak intensity is not linear, the question arises of whether there are losses in the transmission of higher intensities. Figure 9b shows the cumulated intensity of all pixels in each camera image. An almost linear curve is observable. It is also noticeable that the curves for the minimum and maximum bright fibers are very close to each other. This means that the total transmitted intensity is almost identical, although the peak intensity is excessively different. The spots must therefore be of a different shape or size for different bright fibers. As Figure 10 shows, the overall spot sizes differ only slightly, mainly indicating that the spot shape is different. The less bright fiber produces only a slightly larger spot at high intensities. Another finding from Figure 9b is namely that the overall transmitted intensity has only doubled, despite the five-fold increase in the pixel area. Nevertheless, a higher projection intensity has the advantage of shorter exposure, and as a result, more robustness to ambient light and movements during the measurement.

**Figure 11:**
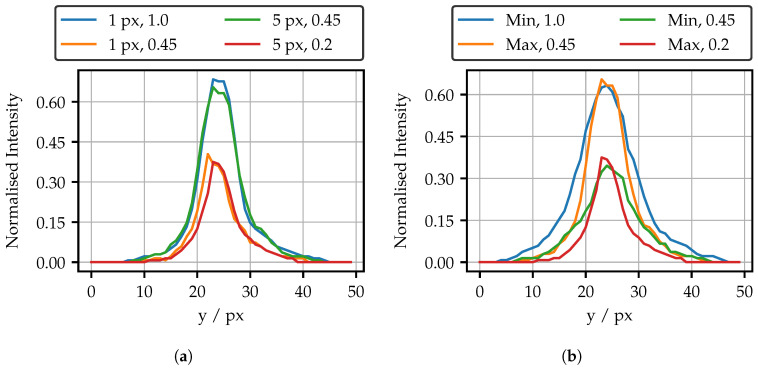
(**a**) Cross sections of the spots of the maximal bright fiber for different projection areas. (**b**) Cross sections of spots of the minimal and maximal bright fiber.

In order to obtain a better idea of the shape of the spots, vertical cross-sections are shown in Figure 11. These include the spots illustrated in Figure 8. On the left are spots with the same peak intensity for the different projection areas of the maximal bright fiber.

For the same peak intensity, approximately twice the modulated intensity is required. Apart from that, the spot shape seems to be independent of the projection area. On the right, the spots of the darkest and the brightest fiber are compared using 5 px projection area per fiber. Spots with the same modulated intensity of 0.45 begin similarly, but separate quickly for higher intensities. For equal peak intensities, the spots of the maximal bright fiber have a much narrower shape.

**Figure 12:**
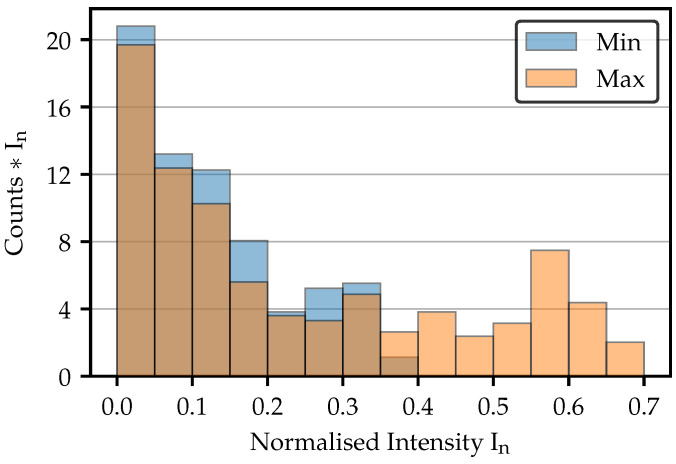
Intensity distribution of the spots illustrated in Figure 8. By weighting the bins with their intensity, the contribution of all intensity values to the total sum is clearly visible. “Min” means minimal bright fiber, and “Max” means maximal bright fiber.

As following from the cumulated intensities in Figure 9b, the spots referring to the cross sections in Figure 11b for a modulated intensity of 0.45 should have a similar cumulated intensity. Due to the overlap of their cross-sections, this is not directly evident. Figure 12 therefore shows the intensity distributions for these spots weighted by the corresponding intensities. The total sums of the bins thus corresponds to the sum of all pixels in Figure 8, as well as to the cumulated intensities in Figure 9b. The distribution clearly shows that the lower intensity values of the darker fiber spot contribute a slightly larger part to the total sum. As a result, the overall sums are similar, despite the much higher maximal peak intensity of the brighter spot.

To sum up this subsection, the main difference between the spots of the minimal and maximal bright fiber is their shape. The total transmitted intensities and the spot sizes differ only slightly. The peak intensities as a function of their modulated projector intensities appear to primarily depend on the maximum achievable intensity of the individual fiber.

### 3.3. Fringe Pattern Adaption

**Figure 13:**
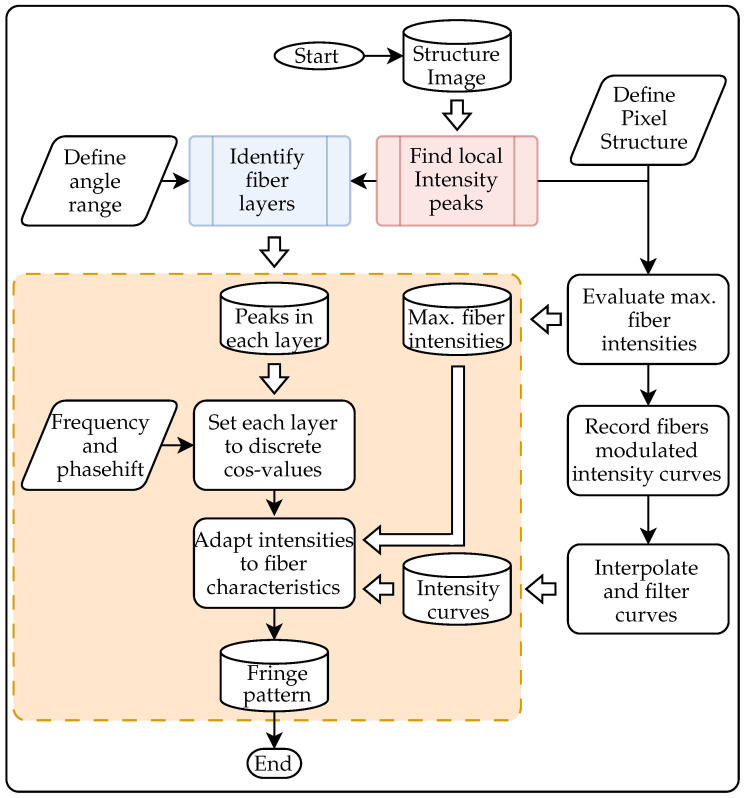
Scheme for the adaption of a fringe pattern. The steps in the orange box are carried out for each pattern, while the other steps are providing the necessary data for the adaption procedure.

With the investigation of the FOIB structure and the characterization of the fiber spots, the basis for the adaptation of the fringe pattern has been created. The adaption algorithm used in this work is shown in Figure 13. The steps required to create new pattern are shown in the orange box. All steps apart from that only need to be carried out once. These include the determination of the fiber centers and the identification of the layers. In addition, the maximal peak intensities of the fibers and the curves of the peak intensities have to be recorded. The peak intensities are determined for each fiber, while the intensity curves are just recorded for around 20 fibers with different maximum peak intensities. As described in the previous subsection, the shape of the intensity curve seems to only depend on the maximum peak intensity. Therefore, the recorded curves are interpolated and filtered as a function of the modulated intensity and the maximum achievable peak intensity. This function is carried out for projection areas of 1 px and 5 px, and enables the adjustment of arbitrary fiber intensities. Once all the required data have been collected, the fiber layers are set to their ideal discrete cosine values depending on the desired frequency and phase shift, and are then adjusted depending on the maximum peak intensity.

**Figure 14:**
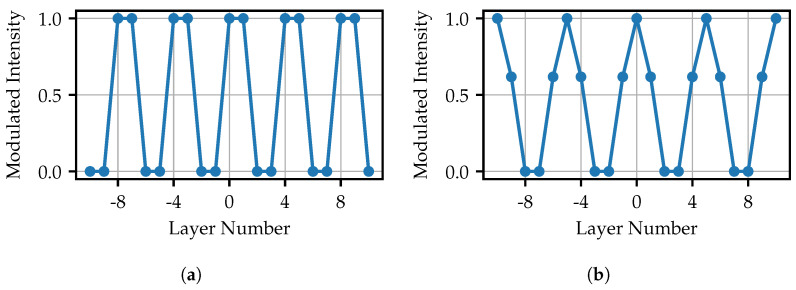
Visualization of the ideal layer values: (**a**) Layer values for approx. 35 and (**b**) for approx. 28 projector amplitude cycles (corresponding to the total projector resolution of 768 px × 768 px).

The visualizations of the ideal modulated values for different frequencies are shown in Figure 14. The central layer is assumed to be the origin. Since no shifts are analyzed in this work, all patterns are starting at this layer.

The highest achievable frequency results from the alternating layers of a modulation of 1 and 0. For all lower frequencies with an odd number of fiber layers, it is more difficult to determine ideal values, as it is not possible to sample both the maximum and the minimum of the cosine function with equidistant steps. In this work, the maxima are scanned exactly. As a consequence, there are always two layers next to the minima. After an adjustment of the dynamic range, the lowest values are set to zero. This definition leads to a better contrast for the projected images. The corresponding function is

(3) $$I_{m}=cos\left(\frac{2\pi }{n_{l}}u\right),$$

where $I_{m}$ is the modulated intensity, $n_{l}$ is the number of layers used in one amplitude cycle, and *u* is the layer number. In that sense, for frequencies with an even number of fiber layers, the cosine function is also discretized such that the sampled points are always located besides the minima:

(4) $$I_{m}=cos\left(\frac{2\pi }{n_{l}}u-\frac{\pi }{n_{l}}\right).$$

**Figure 15:**
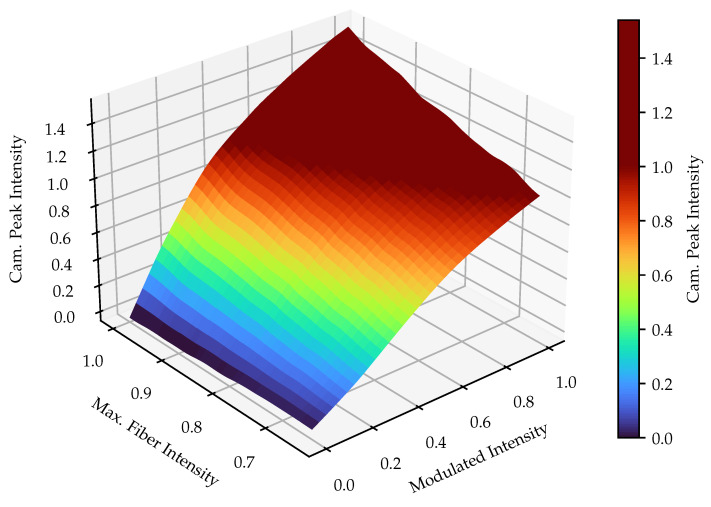
Interpolated and filtered intensity curves for different maximal peak fiber intensities for the adaption of the modulated projector intensity with a projection area of 5 px.

Now that the ideal modulated intensities are known, they have to be adapted to their fibers’ maximal peak intensity. The result of the interpolation over the different intensity curves for the 5 px projection area is shown in Figure 15. Here, all values were normalized to the maximum peak intensity of the darkest fiber. It is not reasonable to use any values greater than one, as they would exceed the maximum possible brightness of the darkest fiber. Therefore, the colormap is scaled to a maximum value of 1.0. A slight ripple is visible at the upper edge, which is caused by the noise in the intensity curves. Apart from that, the correlation between the intensity curves for different maximum peak fiber intensities seems deterministic. In order to adjust the modulated intensity, it is first necessary to determine the intensity curve corresponding to the maximum fiber intensity. Based on the desired camera peak intensity, it is then possible to find the needed modulation intensity.

**Figure 16:**
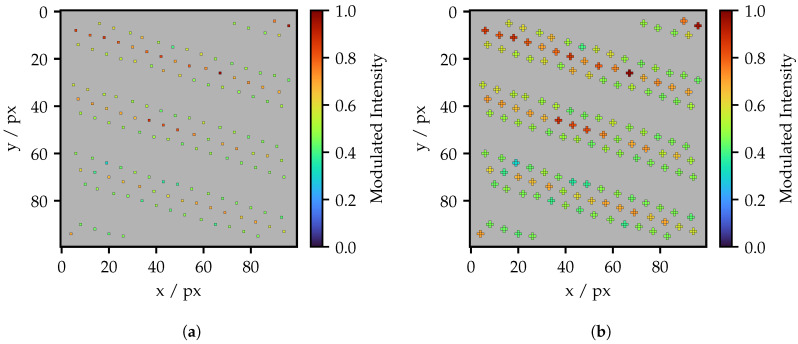
Adapted projector images with 28 projector amplitude cycles with a projection area per fiber of (**a**) 1 px and (**b**) 5 px.

The generated projector image, as an outcome of the adaption process corresponding to Figure 13, is shown in Figure 16. The ideal layer values are equal to Figure 14b. 1 px per fiber is used on the left, 5 px on the right. For better contrast, all pixels with a value of zero are illustrated in gray. It is well recognizable that the middle layers are on average brighter than the two outer layers. The brightness in each layer varies due to the adaption process caused by the different fibers’ maximum peak intensities.

**Figure 17:**
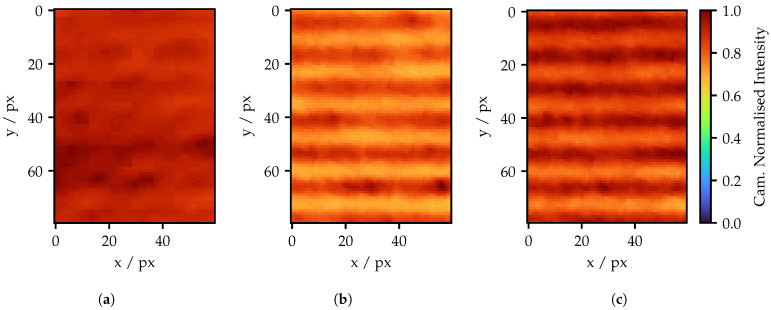
Fringe pattern of (**a**) standard pattern; (**b**) only spatial adapted pattern; and (**c**) spatial and intensity adapted pattern with the highest possible projector frequency of 69 amplitude cycles.

**Figure 18:**
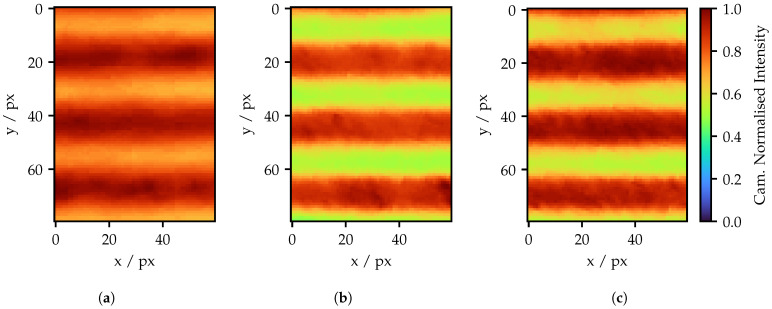
Fringe pattern of (**a**) a standard pattern; (**b**) only a spatial adapted pattern; and (**c**) a spatial and intensity-adapted pattern with a projector frequency of 35 amplitude cycles.

**Figure A3:**
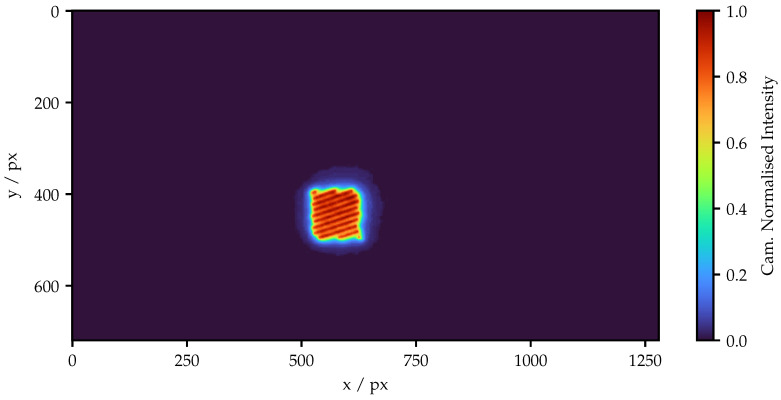
Complete camera image displaying the pattern of the highest possible adapted frequency. The projector ROI and the rotation of the pattern are clearly visible.

Camera image sections of 60 px × 80 px of the adapted fringe pattern for a 5 px projection area are shown in Figure 17 and Figure 18. This section’s size refers to a size on the projection screen of approximately 0.7 mm × 0.9 mm. The highest possible frequency and the third highest frequency are shown, the latter of which corresponds to Figure 16b. For comparison, the unadapted standard fringe pattern is shown, as well as the spatially only adapted pattern. In contrast to the previous subsection, camera exposures are now adjusted, while the LED power stays the same. Since the layers of the FOIB are not horizontally orientated, the fringes are not only rotated in the projector image, but also in the camera image. Appendix Figure A3 shows the full camera image for the highest frequency. Here, the rotation of the fringes is clearly visible. For further analysis, the images of the adapted patterns are each rotated by a fixed angle.

In Figure 17a, almost no fringes are recognizable. A much better result is obtained with the spatially adjusted pattern. The fringes are clearly visible; however, due to the high background brightness, the amplitude is quite small. This is an effect of the spots spread of the individual fibers. As one can see, the pattern repeats in a cycle of about 15 px. The total width of the spots exceeds approximately 40 px according to Figure 8. This means that the amplitude between the maxima cannot drop to zero. Here, the low-pass behavior of the system arises. Compared to the intensity adapted image in Figure 17c, the values in Figure 17b appear smaller overall. This is because single fibers are much brighter and the remaining fibers appear darker due to the normalization. In contrast, the peak values in Figure 17c are more homogeneous, but the variation in spot size is greater, making the edges of the stripes appear unclean. At this point, reference is drawn to Figure 11b, where the narrower spot shape of the brighter fiber for the same peak intensity is visible.

With half the maximum projector frequency, the standard fringe pattern is easily recognizable, as can be seen in Figure 18a. However, the amplitude is significantly lower compared to the adapted patterns. Furthermore, the structure of the fiber layer appears, especially in the lowest stripe. In Figure 18b, individual brighter fibers are apparent due to the lack of intensity adaption. Yet, the effect appears to be lowered by the two adjacent bright fiber layers. In the additional intensity adapted image in Figure 18c, the intensity in the fringes appears much more homogeneous. The edges also appear much cleaner compared to Figure 18a. Nevertheless, these patterns also still suffer from background intensity.

From a subjective assessment, the adaption provides improvement in the pattern quality. In order to quantify this improvement, signal amplitude and noise are calculated over the various frequencies using sections like the ones previously shown. To separate signal and noise, SciPy’s sosfiltfilt function for second-order section (SOS) forward/backward filtering [21]. SOS filters offer high numerical stability, while forward/backward filtering avoid a phase offset in the filtered signal [22]. The challenge in this task is that the noise is largely attributable to the FOIB structure, manifesting primarily within a specific frequency range. For the highest frequency, it is exactly the same frequency range for the signal amplitude. Additionally, it is necessary to filter not only noise but also global brightness gradients, which overlap with lower frequencies. Consequently, the filter needs the capability of effectively separating frequency bands from each other. While a simple mean value filter is primarily suitable for suppressing random noise, an ideal sinc filter introduces noise in the time period [23]. A Butterworth filter, which has moderately fast band separation without a ripple in the pass or stop band, is a good compromise [24].

**Figure 19:**
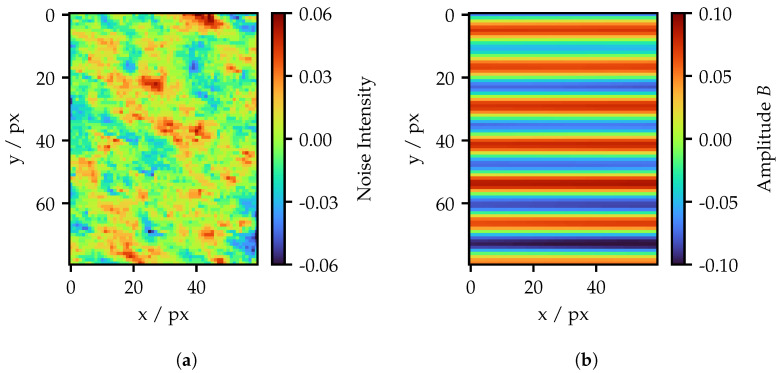
Result of the filtering of Figure 17c. (**a**) Separated noise image and (**b**) separated signal image.

To bypass the problem of the separation of signal and noise, a simple assumption is made: Apart from a global brightness gradient, the intensity values per row should remain constant. Therefore, all the rows of the input image are highpass filtered and recombined into a noise image afterwards, as shown in Figure 19a. To create the signal image, the noise image is first subtracted from the original image. Then, each column is highpass filtered to get rid of any global brightness gradients. The amplitude is calculated by determining the minima and maxima values for each column and averaging them for the whole image. For both filtering operations, a third-order filter is used. The cut-off frequency refers to half a amplitude cycle.

The noise image in Figure 19a shows several minima and maxima. As intended, the rows in Figure 17c containing the maxima show less noise. Yet, the areas between the minima and maxima are sources for the highest noise deviation due to differing spotshapes. Apart from that, there is no recognizable structure and the noise shows a random character. In contrast, the signal image in Figure 19b does not longer show any signs of noise. The only particularity is the slightly lower amplitude in the upper part of the image compared to the lower part. However, this effect is also visible in Figure 17c.

**Figure 20:**
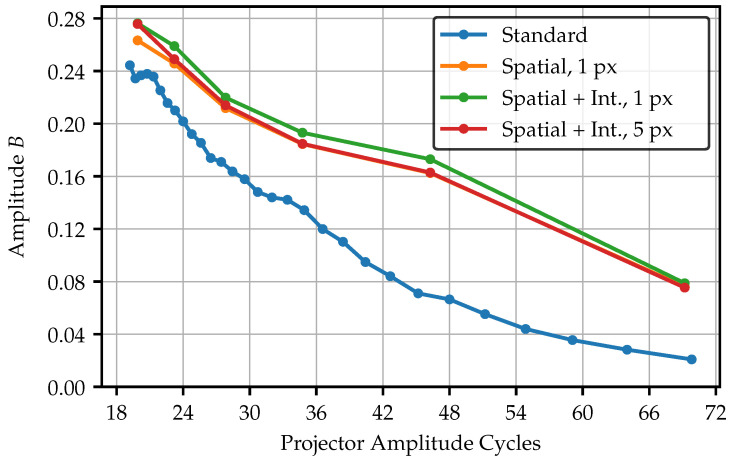
Amplitude for different fringe pattern over a wide frequency range. Due to the limited number of layers, the standard fringes dataset contains more samples.

**Figure 21:**
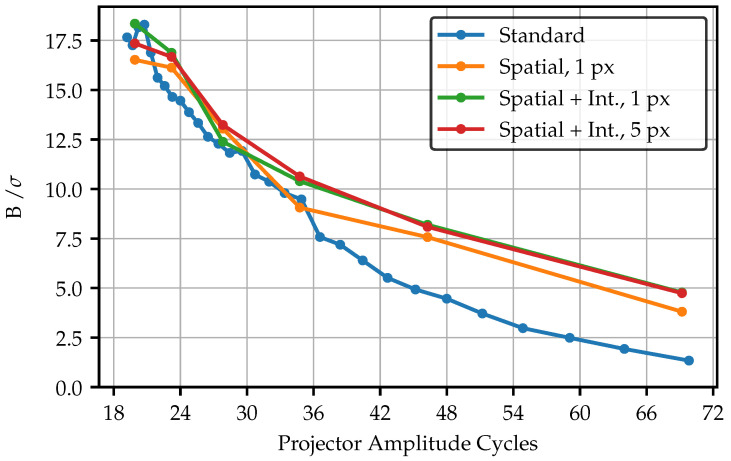
SNR for a different fringe pattern over a wide frequency range. Due to the limited number of layers, the standard fringes dataset contains more samples.

The filtering procedure is now applied to four different image datasets in a projector frequency range from 20 to 70 amplitude cycles (Corresponding to the total projector resolution of 768 px × 768 px). The datasets include unadapted standard fringes, spatially adjusted patterns for 1 px projection area, and a spatially and intensity-adapted pattern for the 1 px, and 5 px projection area. Figure 20 visualizes the amplitude of the signal image across the frequencies while Figure 21 shows the SNR, which results from the ratio of the amplitude and the standard deviation of the corresponding noise image.

Since the adapted patterns are tied to the fiber layers in their frequencies, less sample points are available. Nevertheless, it becomes clear that the adaption of the pattern significantly contributes to an increased amplitude, especially for higher frequencies. In the lower frequency range, the curves begin to converge. Nevertheless, the amplitudes of the adapted patterns remains above the standard pattern, even for the lowest frequency of 20 amplitude cycles. The only spatially adapted pattern with the 1 px projection area performs the best. However, in practical applications, these would not be preferred since the light output is significantly lower and the amplitude only slightly higher compared to the 5 px projection area. Regarding the amplitude, the adaptation of fiber intensities seems to have almost no effect. The amplitude of the intensity adapted 1 px projection area pattern exceeds only slightly better values, caused by absent intensity outliers affecting the normalization.

This is different in case of the SNR. Here, the intensity adapted curves show an improved behavior. Compared to the amplitude, the curves in the SNR start to merge around a frequency of about 36 amplitude cycles. Since the amplitude of the adapted fringes is still much higher at this point, the noise must have increased too. This effect is visible in Figure 18, where the single fibers are more distinctly visible for the adapted fringes, as the image of the standard fringes seems to have a stronger lowpass effect. Nevertheless, the adapted curves remain slightly above the standard patterns. Additionally, the projection screen is located in the best possible focus position. Using a slight defocus on purpose, the SNR could exceed much better values for the same amplitude as the standard pattern. As observed before, no significant differences are visible between the projection areas.

## 4. Discussion

The main hypothesis of this work is proven. By adapting the spatial structure of the pattern and the intensity of individual fibers, it is possible to improve the amplitude and SNR, particularly at higher frequencies. However the curves of the SNR are merging much earlier, indicating that the adaptation also introduces noise created by the FOIB structure. For these frequencies, an increased projector blur at the expense of less amplitude could further improve the SNR. It should therefore be noted that, for a high pattern quality, the system should be precisely positioned. In this context, the spatial adaptation offers a greater advantage than the intensity adaptation does. Nevertheless, the intensity adaption should be done anyway, since the peak intensities of the spots do not behave linearly.

Also, further blurring could be necessary anyway, as binary patterns are used due to the sampling of the cosine function. However, the projection of individual fibers produces Gaussian-like peaks anyway without additional blurring. Asymmetric sampling at high frequencies with an odd number of fiber layers could be more problematic, since the intensity would not behave as perfectly cosine shaped. In any case, further work needs to be undertaken to determine which pattern frequency is best suited for the measurement of defects. As stated in the introduction, not only are SNR and frequency affecting the measurement uncertainty, but so is the number of shifts. The maximum number of shifts possible depends on the number of fiber layers. This means that lower frequencies have the advantage of more shift steps.

Moreover, it should be noted that the proposed algorithm is restricted in the enhancement of the SNR. The fringes are still a superposition of single spots. As a consequence, when perfectly sharp focused, it is not possible to obtain an ideal homogeneous intensity profile. Additionally, the spots themselves fluctuate in their intensity distribution and size, which leads to a difficult adaption process. Despite these factors, the algorithm allows it to use frequencies, which are not possible to transmit without any adaption. But, since the amplitude of these patterns is quite small, the noise introduced by the camera or challenging specimen surfaces exerts a higher impact on the measurement.

A property of the fringe pattern which was disregarded in this work is the fringe direction. When only one direction for the reconstruction is used, there is also an optimal fringe angle [25]. In relation to this work, there are two options for practical applications. First, the FOIB has to be rotated manually. The second possibility is the deployment of all three possible FOIB directions, which would probably improve the phase measurement uncertainty, but also increase the measuring time.

For real-world applications, it is also necessary to improve the speed of the structure identification algorithms, especially of the intensity curve recording. This would enable the measurement on more demanding materials than a paper target, which is characterized by homogeneous reflection. In further studies, the transmission properties of individual fibers will also be investigated in more detail. According to the normalized frequency based on the fibers properties in Table 1, the individual fiber behaves as multimode fibers, whose intensity distribution can vary greatly depending on the light coupling or external factors such as bending or the coherence of the applied light source [26,27,28].

The investigations carried out concerning the fibers’ intensity showed that the sum of the transmitted intensity is almost identical despite different peak intensities. This could also be interpreted to mean that, as the number of fibers increases, the transmission behavior of individual fibers becomes less relevant. In order to avoid nonlinear effects, the highest possible number of fibers is required. The nonlinear effects are also much smaller at low modulation intensities, making them less relevant for simple image transmission.

Using wavefrontshaping, it is possible to control the propagation of light in multimode fibers in a certain manner. For this purpose, DMDs can also be employed [29]. However, the utilization of targeted mode propagation in individual fibers in the context of this work is questionable, as only a few pixels per fiber are available. Nevertheless, more detailed investigations will be carried out in the future. Moreover, it is possible to determine the exact transmission behavior of multimode fibers, and as consequence, obtain images through them or even to generate 3D reconstructions. In comparison to the system presented in this paper, smaller sample sizes in the range of a few to several hundred micrometers are investigated, while the presented system is intended to be used for larger measurement tasks in the range of 5–20 mm [30,31].

## 5. Conclusions

This paper presents methods and results for projecting a high frequency fringe pattern using a fiber optic imaging bundle (FOIB) with a small number of individual fibers by adapting the pattern to the FOIB structure. To do so, in a first step, the spatial structure of the FOIB regarding the projector pixel is identified. At this point the FOIB shows a sufficiently homogeneous structure for adapting the fringes. However, the brightness of different fibers varies. As a consequence, their intensity has to be adapted additionally. After determining the individual fibers center, it is possible to address them directly by the desired pixel structures. In this work, one single and five cross-shaped projector pixels were utilized. As it turns out, the peak values of the spots produced by individual fibers behave nonlinearly as a function of the modulated intensity, which impedes the intensity adaption. However, the total transmitted intensity increases almost linearly. Therefore, it is the shape and intensity distribution within the spot which changes. It was shown that the peak intensities of the spots can be corrected by recording intensity curves. Concerning the projecting pixel area, no difference was found except for a higher light output using more projector pixels.

After determining the layers in the identified hexagonal structure of the FOIB, the desired fringe patterns are generated by specifying the frequency and phase. Applying the aforementioned intensity adaption, the peak values of each projected spot is almost equalized. By computing the amplitude and the signal-to-noise ratio over a wide frequency range, improvements in the patterns quality are verified, particularly at high frequencies. Nevertheless, the amplitude and SNR curves indicate that, for practical application, precise projector blurring could bring further improvements.

Future work will mainly focus on two topics. First, it is now possible to combine the findings of this work with fringe projection profilometry algorithms, to work out the practical application and evaluation for real defects. Secondly, the further characterization of the transmission behavior of the individual fibers can be performed.

## Figures and Tables

**Table 1:** Characteristics of the different FOIBs.

Manufacturer	Type	Fiber Count	Image Circle	Mean Fiber Diameter	NA
Fujikura	Fused	15,000	0.55 mm	3.6 µm	0.39 [19]
Schott	Leached	18,000	1.45 mm	10.8 µm	0.39

## Data Availability

Dataset available on request from the authors.

## Abbreviations

The following abbreviations are used in this manuscript:

FOIB	Fiber Optic Imaging Bundle
DMD	Digital Micromirror Device
LED	Light-Emitting Diode
COG	Center of Gravity
SNR	Signal-to-Noise Ratio
MTF	Modulation Transfer Function
ROI	Region of Interest
SOS	Second-Order Section
